# Ex vivo confocal microscopy for the intraoperative assessment of deep margins in giant basal cell carcinoma

**DOI:** 10.1016/j.jdcr.2022.07.008

**Published:** 2022-07-12

**Authors:** Nebiha Kechrid, Luca Tonellotto, Sandra Monnier, Severin A. Rossi, Franzisca Ulrich, François Kuonen

**Affiliations:** a Department of Dermatology and Venereology, Hôpital de Beaumont, Centre Hospitalier Universitaire Vaudois, Lausanne, Switzerland; b Department of Plastic and Reconstructive Surgery, Centre Hospitalier Universitaire Vaudois, Lausanne, Switzerland

**Keywords:** basal cell carcinoma, confocal microscopy, giant, surgical margins, BCC, basal cell carcinoma, EVCM, ex vivo confocal microscopy

## Introduction

Giant basal cell carcinomas (BCCs) represent a therapeutic challenge as they are associated with a high rate of local recurrence despite optimal therapy.^1^ Standard excision requires wide and often mutilating margins to achieve complete excision.^2^, ^3^, ^4^ Alternatively, Mohs micrographic surgery, regarded as the gold standard for high-risk BCC, offers a more conservative approach, but recurrence rate increases significantly with increasing tumor diameter.^5^^,^^6^ Moreover, Mohs micrographic surgery is extremely time consuming for large aggressive specimens.^2^^,^^6^ Here, we report 2 cases of giant BCCs where conservative removal was guided by intraoperative deep margin assessment using ex vivo confocal microscopy (EVCM) following the procedure described by Grizzetti and Kuonen for Histolog Scanner (SamanTree Medical SA).^7^

## Case report

**Fig 1:**
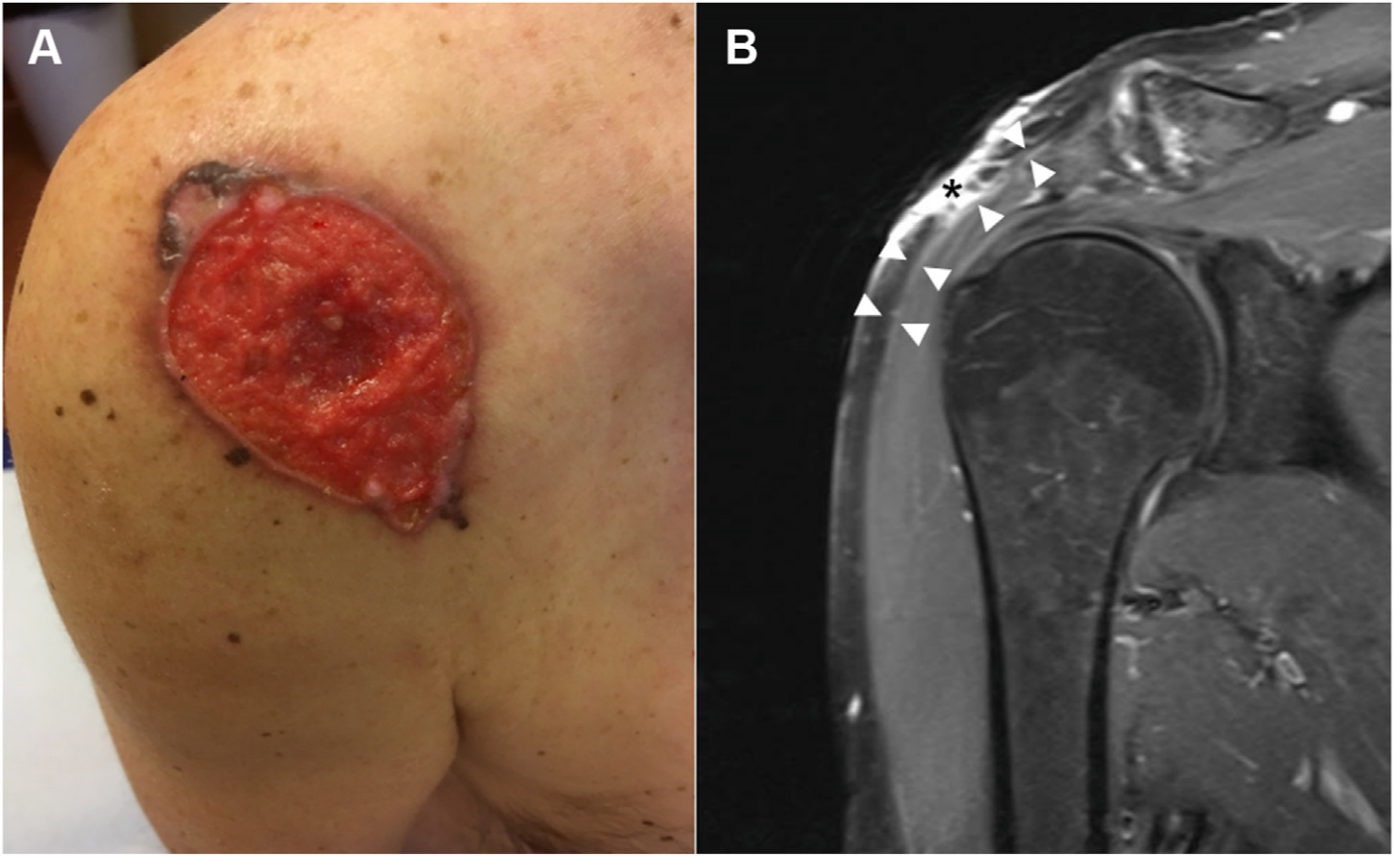
**A,** Clinical picture of a giant basal cell carcinoma (BCC) of the right shoulder. **B,** Sagittal view of a contrast-enhanced T1-weighted magnetic resonance imaging of the right shoulder, showing contrast enhancement in the BCC (*black asterisk*) and in the fascia of the deltoid muscle (delineated by *white arrowheads*).

**Fig 2:**
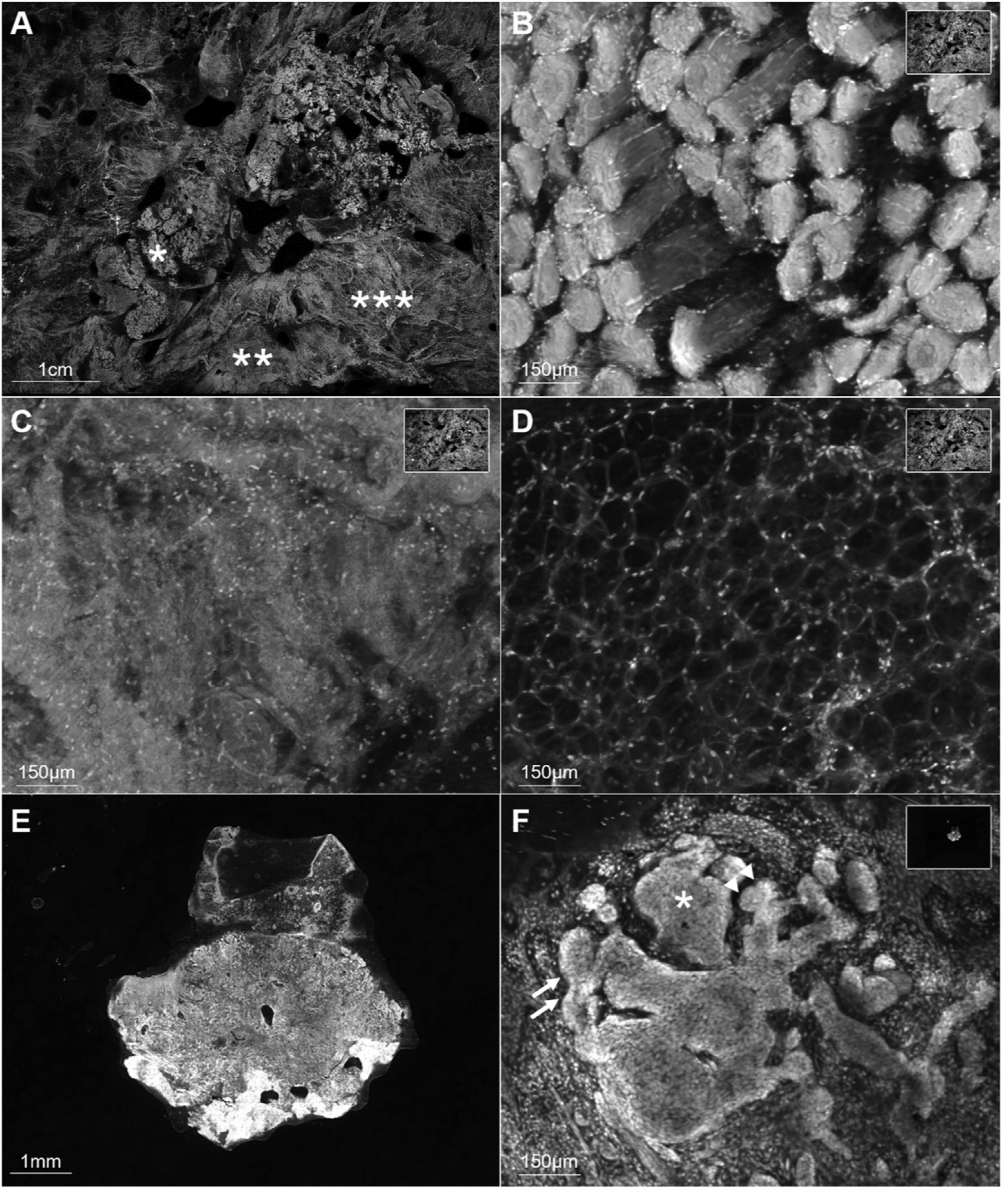
**A,** Illustrative field of view obtained using ex vivo confocal microscopy (EVCM) of the deep surgical margin, composed of muscular (∗), fibroconnective (∗∗), and adipose (∗∗∗) tissues, shown at higher magnifications in (**B**), (**C**) and (**D**), respectively. **E,** Low magnification field of view obtained using EVCM of a punch biopsy taken form the center of the specimen. **F,** High magnification of the punch biopsy shown in (**E**), revealing BCC tumor foci, characterized by nuclear crowding (*asterisk*), peripheral palisading (*arrows*), and clefting (*arrowheads*).

A 58-year-old male presented with a 5-cm nonhealing slowly growing ulcerative lesion located on the right shoulder (Fig 1, *A*). Histopathological examination of a punch biopsy revealed a nodular BCC with deep tissue invasion. Preoperative magnetic resonance imaging confirmed a 56 × 51 × 12 mm lesion in contact with the muscular fascia but without radiological involvement of the underlying deltoid muscle (Fig 1, *B*). Contrast enhancement in the fascia suggested either tumor infiltration or reactive inflammation. We opted for a one-step surgical procedure under general anesthesia with standard wide lateral margins, but conservative deep margins (fascia), to preserve muscular tissue. The entire deep surgical margin (approximately 32 cm^2^) was visualized intraoperatively using EVCM (8 minutes for sample processing, imaging, and analysis) and identified adipose, fibroconnective, and muscular tissues (Fig 2). No tumor foci were detected. The surgical defect was reconstructed during the same procedure using split-thickness skin graft. Ulterior classical histopathological analysis confirmed complete tumor removal. Seventeen months after initial treatment, no recurrence was detected.

**Fig 3:**
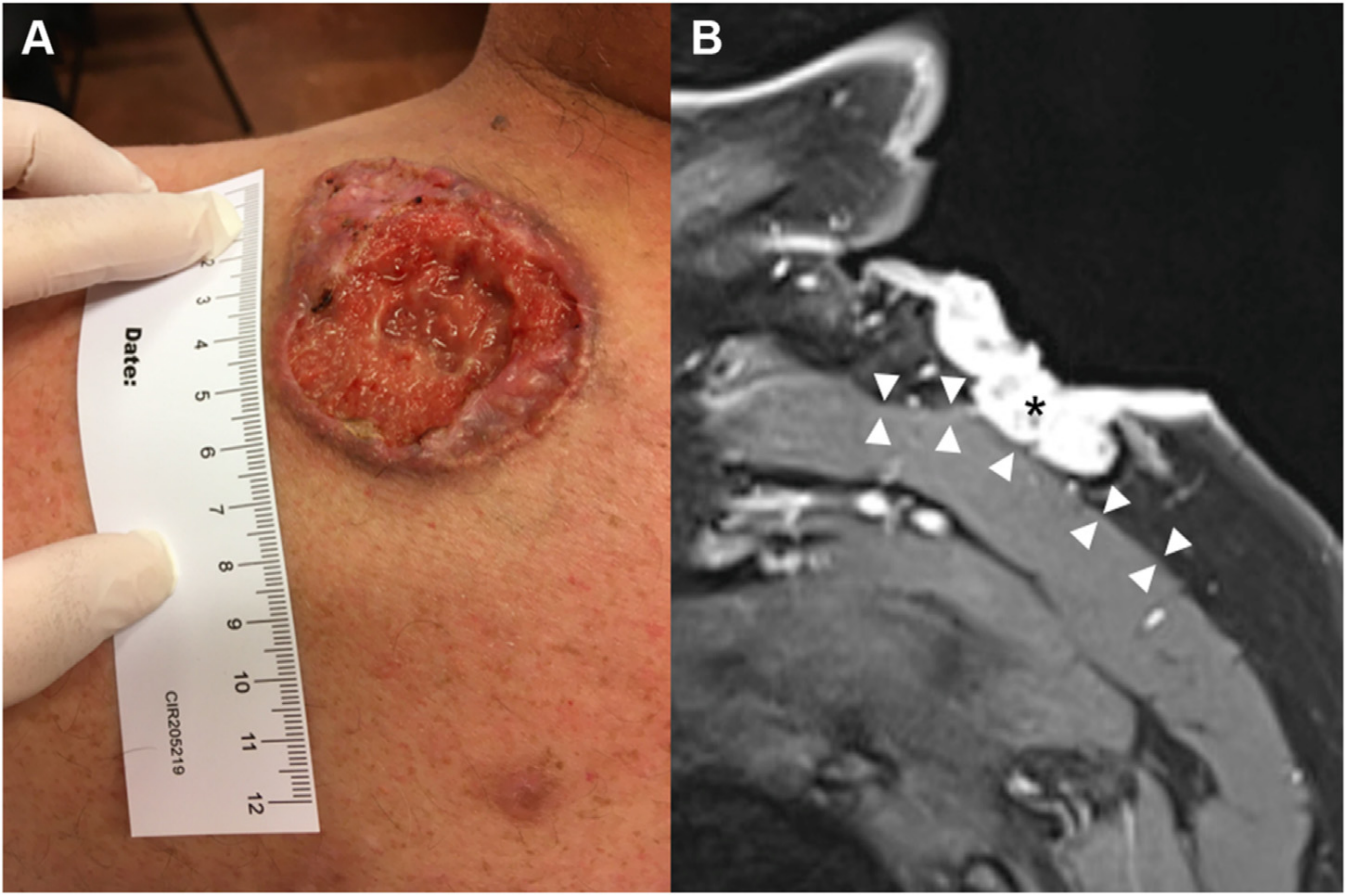
**A,** Clinical picture of a giant basal cell carcinoma (BCC) of the left shoulder. **B,** Sagittal view of a contrast-enhanced T1-weighted magnetic resonance imaging of the left shoulder, showing contrast enhancement in the BCC (*black asterisk*) and in the fascia of the trapezius muscle (delineated by *whitearrowheads*).

**Fig 4:**
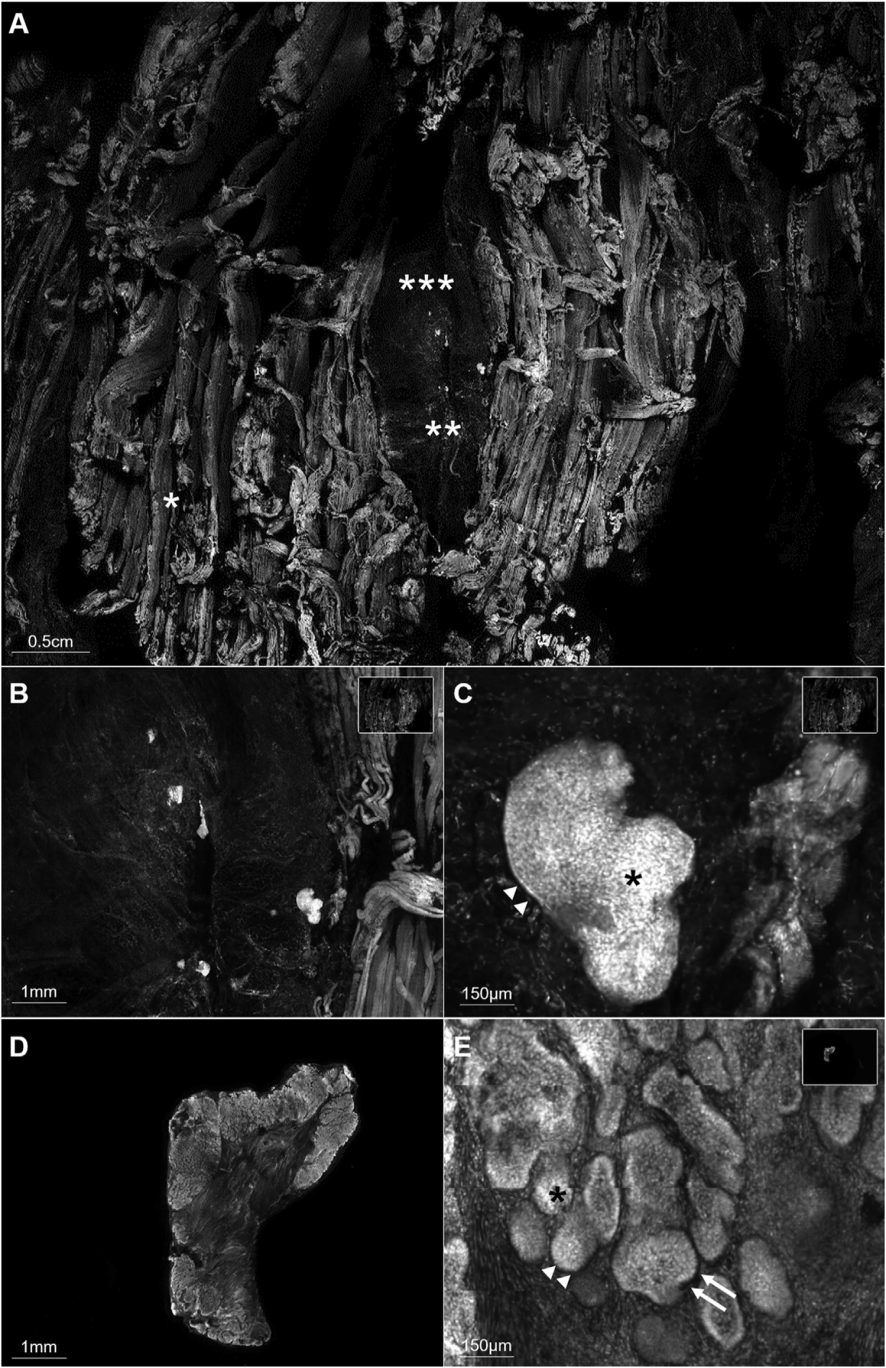
**A,** Illustrative field of view obtained using ex vivo confocal microscopy (EVCM) of the deep surgical margin, composed of muscular (∗), fibroconnective (∗∗), and adipose (∗∗∗) tissues. **B** and **C,** Higher magnifications of the deep surgical margin shown in (**A**) visualized with EVCM showing basal cell carcinoma (BCC) tumor foci embedded in the fibroconnective tissue. **D,** Low magnification field of view obtained using EVCM of a punch biopsy taken form the center of the specimen. **E,** High magnification of the punch biopsy shown in (**D**), revealing BCC tumor foci. Nuclear crowding (*asterisk*), peripheral palisading (*arrows*), and clefting (*arrowheads*) are highlighted in (**C**) and (**E**).

A 52-year-old male presented with a 7-cm, slowly growing tumor on the left shoulder (Fig 3, *A*). Histopathological examination of a punch biopsy revealed a nodular BCC. Preoperative magnetic resonance imaging revealed a 74 × 57 × 14 mm lesion with deep infiltration to the muscular fascia but without radiological involvement of the underlying trapezius muscle (Fig 3, *B*). We opted for a one-step surgical procedure under general anesthesia with standard wide lateral margins but conservative deep margins (superficial muscular layer). The entire deep surgical margin (approximately 69 cm^2^) was visualized intraoperatively using EVCM (20 minutes for sample processing, imaging, and analysis) and identified adipose, fibroconnective, and muscular tissues (Fig 4, *A*), as previously shown. In the center of the deep margin, however, we observed BCC tumor foci invading the muscular fascia (Fig 4, *B-E*). An additional muscle layer was thus removed and the defect reconstructed during the same procedure using split-thickness skin graft. Ulterior classical histopathological analysis confirmed complete tumor removal. Eight months after initial treatment, no recurrence was detected.

## Discussion

Deep local recurrence is particularly problematic as it exposes to delayed detection and deep tissue destruction. EVCM has emerged as a fast and accurate method for the intraoperative assessment of BCC surgical margins.^8^ Peripheral margin assessment may be limited by suboptimal tissue flattening and challenging distinction of BCC foci from adnexal structures in superficial skin layers.^9^ In contrast, deep tissue flexibility (for flattening) and the sharp differentiation of BCC foci from adipose, fibroconnective, or muscular tissues found in the deep subcutaneous layer make EVCM especially suitable for deep margin assessment, as reflected by its excellent negative predictive value.^7^ Because of their rarity, no therapeutic guidelines have been established for giant BCCs yet. Our case reports suggest that intraoperative EVCM may be easily integrated to the therapeutic strategy and improve the outcome of difficult giant BCC, although larger studies with longer follow-ups are required.

## Conflicts of interest

None disclosed.
